# *In Vivo* Correlation of Glucose Metabolism, Cell Density and Microcirculatory Parameters in Patients with Head and Neck Cancer: Initial Results Using Simultaneous PET/MRI

**DOI:** 10.1371/journal.pone.0134749

**Published:** 2015-08-13

**Authors:** Matthias Gawlitza, Sandra Purz, Klaus Kubiessa, Andreas Boehm, Henryk Barthel, Regine Kluge, Thomas Kahn, Osama Sabri, Patrick Stumpp

**Affiliations:** 1 Department of Diagnostic and Interventional Radiology, University Hospital of Leipzig, Liebigstraße 20, 04103 Leipzig, Germany; 2 Department of Nuclear Medicine, University Hospital of Leipzig, Liebigstraße 18, 04103 Leipzig, Germany; 3 ENT-Department, University Hospital of Leipzig, Liebigstraße 10–14, 04103 Leipzig, Germany; Northwestern University Feinberg School of Medicine, UNITED STATES

## Abstract

**Objective:**

To demonstrate the feasibility of simultaneous acquisition of ^18^F-FDG-PET, diffusion-weighted imaging (DWI) and T1-weighted dynamic contrast-enhanced MRI (T1w-DCE) in an integrated simultaneous PET/MRI in patients with head and neck squamous cell cancer (HNSCC) and to investigate possible correlations between these parameters.

**Methods:**

17 patients that had given informed consent (15 male, 2 female) with biopsy-proven HNSCC underwent simultaneous ^18^F-FDG-PET/MRI including DWI and T1w-DCE. SUV_max_, SUV_mean_, ADC_mean_, ADC_min_ and *K* ^trans^, *k*_ep_ and *v*_e_ were measured for each tumour and correlated using Spearman’s ρ.

**Results:**

Significant correlations were observed between SUV_mean_ and *K* ^trans^ (ρ = 0.43; p ≤ 0.05); SUV_mean_ and *k*_ep_ (ρ = 0.44; p ≤ 0.05); *K* ^trans^ and *k*_ep_ (ρ = 0.53; p ≤ 0.05); and between *k*_ep_ and *v*_e_ (ρ = -0.74; p ≤ 0.01). There was a trend towards statistical significance when correlating SUV_max_ and ADC_min_ (ρ = -0.35; p = 0.08); SUV_max_ and *K* ^trans^ (ρ = 0.37; p = 0.07); SUV_max_ and *k*_ep_ (ρ = 0.39; p = 0.06); and ADC_mean_ and *v*_e_ (ρ = 0.4; p = 0.06).

**Conclusion:**

Simultaneous ^18^F-FDG-PET/MRI including DWI and T1w-DCE in patients with HNSCC is feasible and allows depiction of complex interactions between glucose metabolism, microcirculatory parameters and cellular density.

## Introduction

^18^Fluor-fluorodesoxyglucose positron emission tomography combined with magnetic resonance imaging (^18^F-FDG-PET/MRI) seems to be a promising modality for imaging of head and neck squamous cell carcinoma (HNSCC). In this type of malignancy an accurate diagnosis of infiltration of surrounding structures is important for local staging and for surgical and radiotherapy planning [1–4]. With the high soft-tissue contrast of MRI and the superior ability of ^18^F-FDG-PET to detect vital tumor tissue prior to morphological changes, the advent of combined PET/MRI will open new perspectives in non-invasive imaging [3]. The combination of PET with MRI also opens up options to acquire multimodal molecular imaging parameters simultaneously. This may contribute to a more detailed characterization of molecular processes *in vivo* [5]. We report about the first study in which glucose metabolism (assessed by ^18^F-FDG-PET), tumor cellularity (measured by diffusion-weighted imaging, DWI) and microcirculatory parameters (estimated by T1-weighted dynamic contrast-enhanced MRI, T1w-DCE) were simultaneously acquired in patients with HNSCC. Not only are these parameters known to be correlated with molecular markers for angiogenesis, proliferation or cell density [6–8]; first and foremost they are of special interest for prediction of patient outcome and response to chemotherapy or combined radiochemotherapy. For the future, the combination of these parameters may further facilitate treatment planning and prognostic stratification [9–12].

## Materials and Methods

### Patients

**Fig 1:**
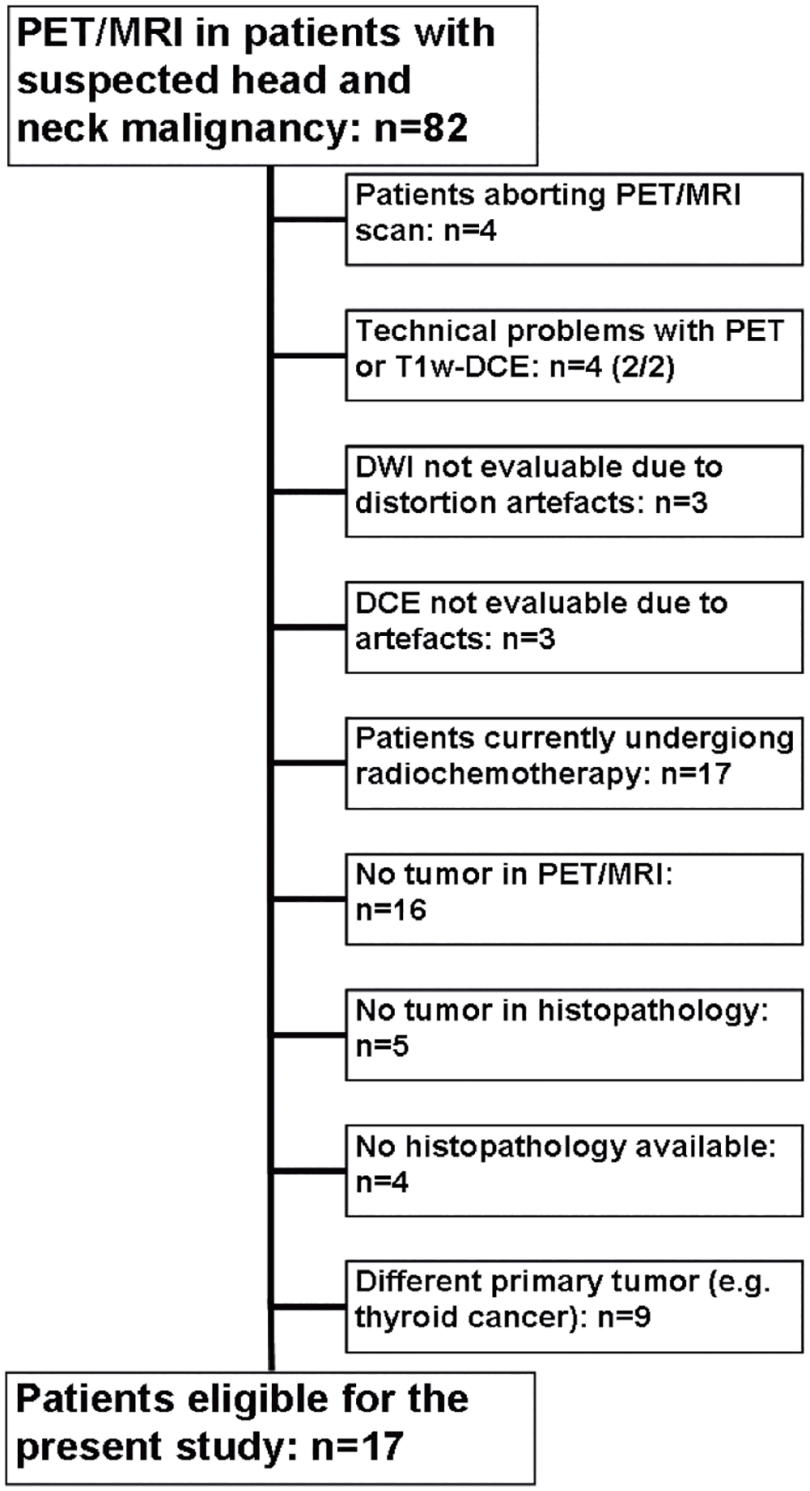
Flowchart of the study population.

From October 2011 to September 2013, 82 consecutive patients with suspected malignancy of the head and neck or a cancer of unknown primary with cervical lymphadenopathy were scheduled to undergo ^18^F-FDG-PET computed-tomography (^18^F-FDG-PET/CT) for staging and treatment planning and, without additional radiopharmaceutical administration, an integrated simultaneous PET/MRI study. This study was IRB-approved and all patients gave their written informed consent. Patients were retrospectively included in the current study if they fulfilled the following inclusion criteria: (a) if a de-novo or recurring HNSCC of the upper aerodigestive tract was histopathologically proven either by biopsy or by resection after imaging, (b) if a histopathological report was available for a specimen taken from the area that was suspicious for tumor in imaging, (c) if a dedicated simultaneous PET/MRI of the neck including T1w-DCE and DWI sequences was performed with sufficient image quality not distorted by motion artefacts; (d) if a tumor was delineable in the imaging studies and (e) if there was no ongoing (radio)chemotherapy. Altogether, 17 patients fulfilled all inclusion criteria (see Fig 1).

### Imaging preparation

All 17 patients underwent an ^18^F-FDG-PET/CT protocol on a Siemens Biograph 16 PET/CT scanner and a simultaneous whole-body PET/MR after a fasting period of at least 6 hours. In 15 of 17 patients PET/CT was performed first and then PET/MRI, in two patients PET/MRI was performed prior to PET/CT due to logistic reasons. Administration of ^18^F-FDG was performed once for both studies, depending on body weight (5 MBq/kg, range 205–396 MBq). Postinjection median uptake time was 83 minutes (range 60–120 minutes) for the first imaging study and 196 minutes (range 150–260 minutes) for the second imaging study.

### PET/MRI scanner

All examinations were performed on a simultaneous PET/MRI scanner (Siemens Biograph mMR; Siemens Healthcare, Erlangen, Germany). The system is composed of a PET detector ring mounted into a 3.0T whole-body magnetic resonance tomograph, resulting in a hybrid imager with a bore diameter of 60 cm and a magnet length of 163 cm. Maximum gradient strength accounts for 45 mT/m, slew rate for 200 T/m/s in all three spatial directions. The MRI-compatible PET detector ring is implemented inside the bore and consists of 56 LSO-APD (lutetium oxyorthosilicate scintillation crystals combined with avalanche photodiodes) block detectors with 64 detector ring elements arranged on the z-axis. This yields an axial field of view (FOV) of about 60 cm and a FOV of about 26 cm in z-direction. Maximum scan length is about 160 cm without repositioning. More detailed descriptions of the technical aspects were described in previous publications [13,14].

### Simultaneous PET/MRI imaging protocol

Patients were placed in supine position with their arms beside the trunk. PET/MRI was conducted in two steps. First, whole-body imaging without contrast medium was performed in six bed positions (head, neck, thorax, abdomen, pelvis and proximal thighs) with a coronal 3D-encoded gradient-echo sequence for attenuation correction (Dixon-VIBE) followed by coronal T2-weighted turbo inversion recovery magnitude (TIRM) and axial T2-weighted half Fourier single shot turbo spin echo (HASTE) sequences as well as axial echoplanar imaging diffusion weighted images (EPI-DWI) with b-values of 0 and 800. Simultaneous to MRI, PET image acquisition was conducted with 5 minutes of scan time per bed position.

Afterwards, a dedicated PET/MRI of the neck using a combined head and neck coil was performed, which also included a coronal Dixon-VIBE sequence for attenuation correction. This was followed by axial T1-weighted turbo spin echo (TSE) and T2-weighted TSE sequences with fat suppression, a coronal T2-weighted TIRM and an axial DWI-EPI sequence with b-values of 0 and 800 (TR/TE 8620/73 ms, slice thickness 4 mm, voxel size 3.2 x 2.6 x 4.0 mm³). Dynamic contrast-enhanced imaging was performed during the administration of 0.1 mmol Gadobutrol per kg of bodyweight (Gadovist, Bayer Healthcare, Leverkusen, Germany) at a rate of 3 ml per second and flushing with 10 ml of normal saline using a power injector (Spectris Solaris, Medrad/Bayer Healthcare, Leverkusen, Germany). T1w-DCE consisted of 40 subsequent scans with a duration of 6 seconds (40 slices per scan), a TR/TE of 2.47/0.97 ms, a slice thickness of 5 mm, a flip angle of 8° and a voxel size of 1.2 x 1.0 x 5.0 mm³; the contrast application was started after the fifth scan. Furthermore axial and coronal fat saturated T1-weighted TSE sequences and an axial contrast enhanced T1-weighted VIBE sequence were conducted after contrast application. Altogether, the dedicated neck protocol accounts for about 30 min of scan time, during which dedicated PET of the neck was conducted for 10 minutes. PET images were reconstructed using the iterative ordered subset expectation maximization algorithm with 3 iterations and 21 subsets, a Gaussian filter with 3 mm full width at half maximum (FWHM), and a 256 x 256 image matrix. Attenuation correction of the PET data was performed using a four-tissue (fat, soft tissue, air, background) model attenuation map which was generated from a Dixon-Vibe MR sequence. A dedicated description of a typical imaging protocol including the complete set of sequence parameters was published recently [1].

### Image analysis

PET data sets were reviewed on a commercially available workstation (using Syngo.Via, Siemens Healthcare, Erlangen, Germany) by one resident in diagnostic radiology with 4 years and one board certified nuclear medicine physician with 7 years of experience in head and neck CT, MR and PET/CT imaging. For all tumors, mean and maximum standardized uptake values (SUV) were analyzed in the PET dataset of the neck with the nuclear medicine physician plotting an isocontour volume of interest (VOI) around the tumor (SUV_max_ threshold 40%).

T1w-DCE images were processed with a commercially available software module for tissue perfusion estimation (Tissue 4D, Siemens Medical Systems, Erlangen, Germany) as described previously [15]. The software offers a population based approach for the arterial input function (AIF) and the best of three available AIF-options was chosen according to the result of the chi2-parameter, which serves as an error measure for the model fit. After scaling the AIF in relation to the gadolinium dose and modelling it by the most widely used bi-exponential model by Tofts and Kermode [16], the pharmacokinetic parameters *K* ^trans^, *k*_ep_ and *v*_e_ were calculated. In this two-compartment model, the volume transfer constant *K* ^trans^ estimates the diffusion of contrast medium from the plasma through the vessel wall into the interstitial space, thus representing vessel permeability. *v*_e_ expresses the volume of the extravascular extracellular leakage space (EES). *k*_ep_ is a parameter for diffusion of contrast medium from the EES back to the plasma. It is in close relation with *K* ^trans^ and *v*_e_ and is calculated by the formula *k*_ep_ = *K* ^trans^ x *v*_e_^-1^. The basic concept of these parameters and their application in patients with HNSCC is described in greater detail in other publications [15,17]. For each patient, these four parameter maps were projected onto the T2-weighted fat-suppressed TSE sequences and the radiologist delineated the tumor manually on each slice, resulting in mean values of *K* ^trans^, *k*_ep_ and *v*_e_ averaged over the complete tumor. Attention was paid not to include areas of gross necrosis or large feeding vessels in close proximity into the ROI.

**Fig 2:**
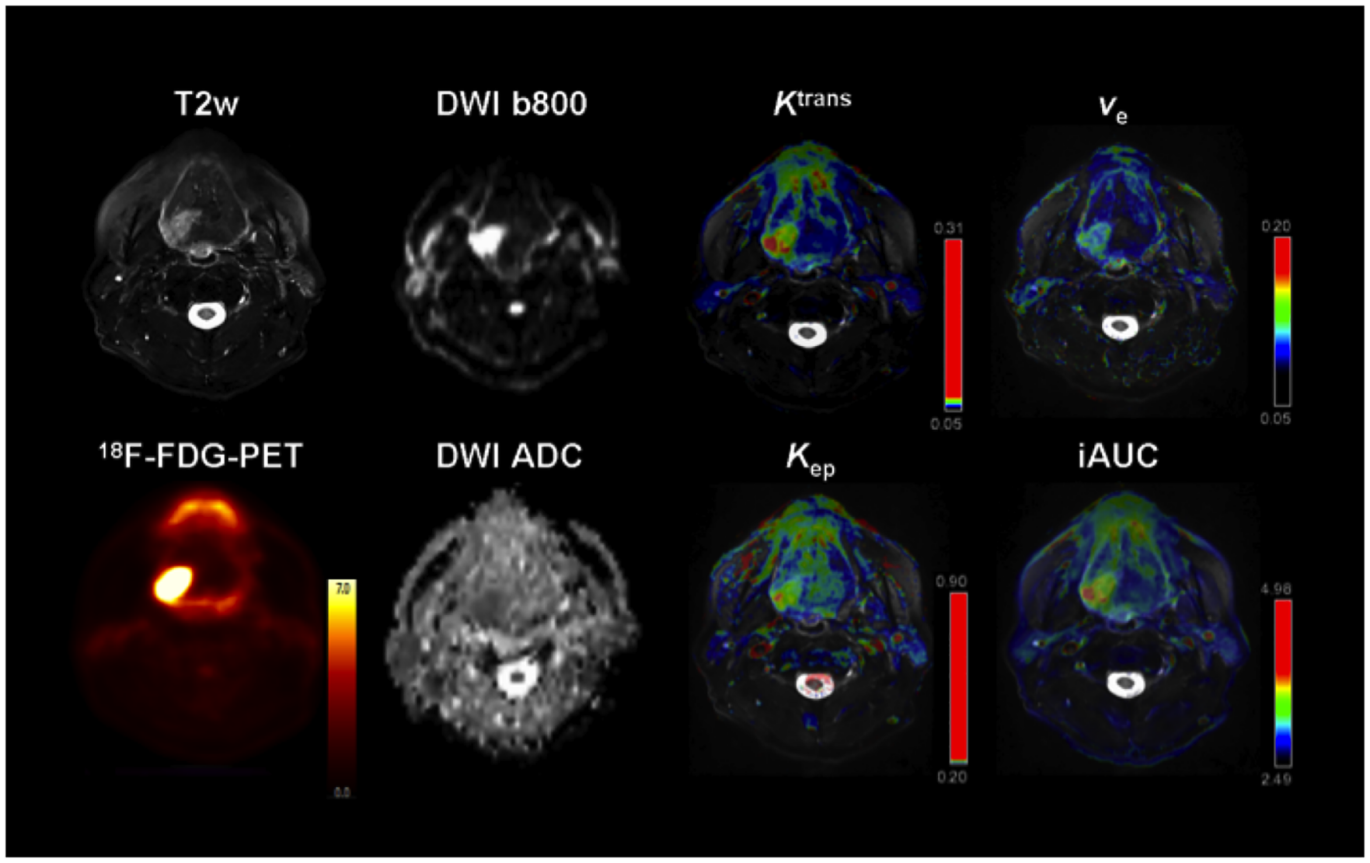
Example of a 59-year old male patient depicting the different molecular parameters obtained by simultaneous PET/MRI. Note the biopsy-proven secondary squamous cell carcinoma of the base of the tongue on the right side. 23 months ago the patient was operated on a squamous cell carcinoma of the soft palate on the same side.

DWI images were transferred to a desktop computer with Mac OS X (Apple, Cupertino, California, USA) and an open-source freeware 4D DICOM viewer (OsiriX, Pixmeo, Geneva Switzerland) [18]. ROI’s were manually drawn on the apparent diffusion coefficient (ADC) maps along the contours of the tumor on each slice in cognitive fusion with the complete MRI and PET datasets; mean and minimal ADC values (ADC_mean_ and ADC_min_) were then averaged for the whole tumor volume. Again, necrotic tumor areas were not to be included into the ROI. An example of the combined molecular parameter maps is depicted in Fig 2.

### Statistical analysis

Statistical analysis and graphics creation was performed with SPSS 20 (IBM SPSS Statistics, Armonk, New York, USA). Values are presented as mean ± standard deviation (SD). Mean value comparison was carried out using the Mann-Whitney-U test. Spearman’s non-parametric rank sum correlation coefficients were calculated between DCE parameters, SUV_max_, SUV_mean_, ADC_mean_ and ADC_min_. Significance level was set at p ≤ 0.05.

## Results

Of the 17 patients, 15 were male and 2 female. Mean age was 57.7 ± 7.3 years (range 49–79 years). Tumors were 11 primary cancers and 6 recurrent cancers, located in the oral cavity (n = 4), in the oropharynx (n = 8) or in the hypopharynx and larynx (n = 5). In patients with recurrent HNSCC, mean time from the end of therapy to diagnosis of the recurring carcinoma was 46 months (range from 12 to 120 months). Patient and tumor characteristics are shown in Table 1, functional imaging parameters of our patient group are depicted in Table 2.

**Table 1:** Patient and Tumor characteristics.

Patient characteristics				Tumor characteristics						
Patient number	Sex	Age (yrs.)	Primary or recurrent cancer	Tumor site	Tumor volume in PET (cm³)	Tumor size in MRI (cmxcm)	Tumor staging(original staging for recurrent cancers)			Tumor grading
1	M	54	primary	Oropharynx	18,0	3,8 x 3,2	cT3	cN2b	cM0	G2
2	M	57	primary	Oropharynx	3,3	2,4 x 1,6	cT2	cN3	cM0	G2
3	M	50	primary	Hypopharynx	12,6	3,4 x 3,3	cT4b	cN3	cM0	G2
4	M	56	primary	Hypopharynx	4,2	1,9 x 1,6	cT3	cN1	cM0	G3
5	M	61	primary	Larynx	8,0	3,5 x 2,6	pT3	pN2c	cM0	G3
6	F	66	primary	Oropharynx	9,5	3,0 x 2,4	cT2	cN2b	cM0	G3
7	M	49	primary	Hypopharynx	22,1	4,2 x 2,5	cT4	cN2c	cM0	G2
8	M	50	primary	Oro-/ Hypopharynx	42,7	3,8 x 3,2	cT4b	cN2b	cM1	G2
9	F	57	primary	Tongue	5,9	3,6 x 1,7	cT4a	cN2c	cM0	G1
10	M	54	primary	Oropharynx	6,9	3,8 x 2,7	cT3	cN2c	cM0	G3
11	M	63	primary	Oro-/ Hypopharynx	17,3	5,0 x 2,3	pT3	pN3	cM0	G3
12	M	59	recurrent	Tongue	4,1	2,3 x 1,4	cT3	cN1	cM0	G2
13	M	59	recurrent	Tongue	13,1	3,4 x 1,4	cT4a	cN2c	cM0	G2
14	M	79	recurrent	Oral diaphragm	44,1	4,7 x 3,2	pT1	pN0	M0	G3
15	M	53	recurrent	Oropharynx	5,1	2,5 x 0,9	pT3	pN0	M0	G3
16	M	52	recurrent	Larynx	2,9	1,7 x 1,0	pT4a	pN2c	M0	G3
17	M	61	recurrent	Tongue / Oral diaphragm	10,6	2,3 x 1,8	pT3	pN0	M0	G2

**Table 2:** Functional imaging parameters.

Parameter	All patients (n = 17)
SUV_max_	20.4 ± 7.78
SUV_mean_	12.3 ± 5.07
ADC_mean_ (mm^2^/s)	1287 ± 150
ADC_min_ (mm^2^/s)	659 ± 175
*K* ^trans^ (min^-1^)	0.19 ± 0.06
*k*_ep_ (min^-1^)	0.41 ± 0.18
*v*_e_	0.53 ± 0.13

**Fig 3:**
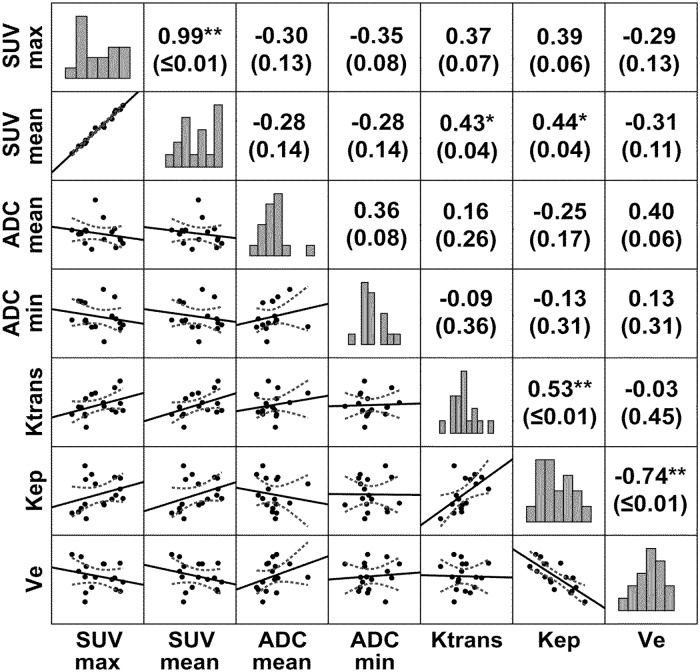
Correlation map of the different molecular imaging parameters in HNSCC. Correlations between ^18^F-FDG-PET, T1w-DCE and DWI were computed using Spearman’s non-parametric rank sum correlation coefficient. Numbers in parentheses represent p-values. * p≤ 0.05; ** p≤ 0.01. Bar graphs indicate the distribution of values.

Significant correlations were observed between SUV_mean_ and *K* ^trans^, and between SUV_mean_ and *k*_ep_. Significant correlations were also present between the microcirculatory parameters *K* ^trans^ and *k*_ep_, and between *k*_ep_ and *v*_e_. Furthermore we noted a trend towards an inverse correlation between SUV_max_ and ADC_min_ and a trend and towards a positive correlation between SUV_max_ and the DCE parameters *K* ^trans^ and *k*_ep_. Also between ADC_mean_ and *v*_e_ a trend towards a positive correlation was apparent. Results are depicted in Fig 3.

## Discussion

Recently, a study on patients with suspected cancer of the head and neck region undergoing simultaneous ^18^F-FDG-PET/MRI after routine ^18^F-FDG-PET/CT was published in which no statistically significant differences regarding sensitivity, specificity, PPV and NPV was found between the two hybrid imaging modalities [1]. The current study demonstrates that the *in vivo* assessment of glucose metabolism, tissue cell density and microcirculatory parameters of HNSCC is feasible with simultaneous PET/MRI. At this point it has to be acknowledged that these analyses can also be performed on sequentially obtained data. However, this work demonstrates that PET/MRI can display complex interactions between glucose metabolism and microcirculation (expressed by correlations between SUV and *K* ^trans^ / *k*_ep_), between glucose uptake and cellular density (depicted by correlations between SUV and ADC) and between cellularity and volume of the extravascular space (estimated by the correlation between ADC and *v*_e_. As all correlations between the different molecular modalities were at best moderate, their combined acquisition seems to provide complementary and not redundant information; yet, they seem to be connected to a certain degree.

We observed a significant correlation and a trend towards a correlation, respectively, between SUV_mean_/SUV_max_ and *K* ^trans^. This can be interpreted as an indicator of an elevated neoangiogenesis in tumors with a higher proliferation rate and a higher glucose demand. In these tumors the expression of vascular growth factors might lead to the formation of a primitive vascular plexus. This primitive vascular plexus typically shows an increased leakiness which is represented by the transfer constant *K* ^trans^ in the Tofts model [15]. Our results support the theory of other studies in which relations between the vessel permeability measured by T1w-DCE MRI and the expression of vascular endothelial growth factor were observed in colorectal and breast cancer [19,20]. Furthermore, the correlation between glucose metabolism measured by ^18^F-FDG-PET and vascular endothelial growth factor (VEGF) expression has already been proven for example in oesophageal squamous cell cancer [21]. After leakage into the tumor’s extravascular space, the blood and contrast medium have to be transported back into the plasma, which is expressed by the rate constant *k*_ep_. As the latter is closely related to *K* ^trans^, also its correlation with glucose uptake seems logical. This also supports a previous study in which a correlation between *k*_ep_ and the FDG dose uptake ratio was reported in patients with breast cancer [22]. For HNSCC Bisdas *et al*. used CT-perfusion and showed a positive correlation between glucose metabolism and vessel permeability (PS), which is essentially akin to *k*_ep_ [23]. Nevertheless there are ambiguous results described in the literature concerning the relation between glucose metabolism and vessel permeability estimated by *k*_ep_ or *K* ^trans^. In several other studies on HNSCC no correlations between SUV_max_ and *k*_ep_ and / or *K* ^trans^ were detected [8,15]. In a study on hepatocellular carcinomas the authors even reported on an inverse correlation between SUV values and *K* ^trans^ [24].

In our study a positive correlation was observed between *K* ^trans^ and *k*_ep_. This positive correlation was also highlighted in a previous examination of patients with HNSCC using T1w-DCE [15]. That study concluded that the enlarged fenestrae of the immature neovessels, which promote contrast medium extravasation (measured by *K* ^trans^) in turn also allow for a fast influx back into the capillary plasma, which is estimated by *k*_ep_[15]. Moreover, these two parameters are linked by the aforementioned formula *k*_ep_ = *K* ^trans^ x *v*_e_^-1^. Another parameter that can be obtained from T1w-DCE analysis is iAUC, the area under the curve. Yet, the interpretation of the correlations for this parameter is difficult and should be treated with caution as iAUC itself is a model-free parameter and is as such prone to variations caused by the length of an acquired T1w-DCE dataset or by different physiological conditions. It was therefore stated by Cheng [25] that conventional iAUC could not be used as a surrogate pharmacokinetic parameter and that pharmacokinetic modelling (e.g. Tofts’ and Kermode’s model [16]) might be the “ideal approach” for accurate quantification—if several conditions, like a valid AIF, are met. This is also why we decided to exclude iAUC from our analysis.

The strong negative correlation between *k*_ep_ and *v*_e_ supports the results of previous works by Bisdas *et al*. [15] and Jansen *et al*. [8] in which similarly strong inverse correlations between these two parameters were observed. The authors ascribed this finding to the smaller interstitial space (expressed by *v*_e_) being responsible for a higher back-flux rate into the plasma (represented by *k*_ep_) because diffusion of a molecule usually happens from a region of higher concentration to one with a lower concentration.

The trend towards a positive correlation between ADC_mean_ and *v*_e_ that we observed also seems logical. In a tumor with less dense cell complexes the ADC values increase as a measure of less restricted water diffusion; smaller cell density should in turn also result in a larger extravascular space, measured by *v*_e_. Yet, this could not be proven in patients with neoplasms of the brain [26–28] or the breast [29]. Our results therefore give a hint that this assumption might actually be true, at least with regards to HNSCC.

Concerning the associations between SUV_max_ and ADC_min_ we observed a trend towards a weak to moderate inverse correlation in our study. As opposed to the relation between T1w-DCE and DWI and that between T1w-DCE and PET, which have been subject to little research to date, publications about the correlation between ^18^F-FDG-PET and DWI parameters are numerous and their results partly ambiguous. Since glucose metabolism is known to be positively correlated with the amount of viable tumor cells and growth rate, an inverse correlation between FDG uptake and ADC values, which reflect tumor cellularity, should be expected [7,30]. Nevertheless, whereas this presumption was confirmed for example in rectal [31], cervical [32], lung [33,34] and breast cancer [35], a similar relation could be demonstrated for HNSCC in only one publication [12] whereas in several other studies no such correlation was apparent [36–38]. The trend towards a moderate inverse correlation between SUV_max_ and ADC_min_ in our patient group might indicate that these parameters are not completely independent and support the hypothesis of Nakajo *et al*. who concluded that the glycolytic activity of HNSCC seems to be partly related with their microstructural environment [12].

In the future, simultaneous functional imaging with PET/MRI could be of special interest for treatment planning and prognostic stratification of oncologic patients. DWI and T1-DCE as well as ^18^F-FDG-PET were proven to be suitable for this purpose in patients with HNSCC prior to radiochemotherapy [9–12]; a satisfactory therapy response and a better prognosis is thought to be related to higher *K* ^trans^ [9], higher ADC_mean_ and lower SUV_max_ values [12]. During successful radiochemotherapy ADC values are increasing [39], whereas ^18^F-FDG uptake and *K* ^trans^ are known to decrease as a sign to therapy response [40,41]. With PET/MRI and a combined acquisition of these parameters further studies to investigate the most suitable modality for assessment and prediction of therapy response are possible.

Our study has several limitations with its small patient number and its retrospective design being the most important ones. The high exclusion rate attributable to technical reasons shows that such sophisticated examinations are probably not yet ready for clinical routine imaging. As it was a pilot study, our results have to be proven for larger patient series. Higher patient numbers could also compensate for the partial lack of statistical significance. As we only studied the tumors themselves and not lymph nodes, on-going studies are focusing on the question to which extent the results are adaptable to nodal metastases in HNSCC.

## Conclusion

Simultaneous ^18^F-FDG-PET/MRI including DWI and T1w-DCE in patients with HNSCC is feasible and allows depiction of complex interactions between glucose metabolism, microcirculatory parameters and cellular density; in the future this might contribute to the planning and adaptation of treatment plans with the aim of optimizing patient outcomes.
